# Team performance during vacuum-assisted vaginal delivery: video review of obstetric multidisciplinary teams

**DOI:** 10.3389/fmed.2024.1330457

**Published:** 2024-03-20

**Authors:** L. Brogaard, L. Rosvig, K. R. Hjorth-Hansen, L. Hvidman, K. Hinshaw, O. Kierkegaard, N. Uldbjerg, T. Manser

**Affiliations:** ^1^Department of Obstetrics and Gynecology, Aarhus University Hospital, Aarhus, Denmark; ^2^Department of Clinical Medicine, Aarhus University, Aarhus, Denmark; ^3^Department of Obstetrics and Gynecology, Randers Hospital, Randers, Denmark; ^4^Department of Oncology, Aarhus University Hospital, Aarhus, Denmark; ^5^Department of Obstetrics and Gynecology, Sunderland Royal Hospital, Sunderland, United Kingdom; ^6^Department of Obstetrics and Gynecology, Horsens Regional Hospital, Horsens, Denmark; ^7^FHNW School of Applied Psychology, University of Applied Sciences and Arts Northwestern Switzerland, Olten, Switzerland

**Keywords:** performance, obstetric, labor, vacuum extraction, video, teamwork, checklist

## Abstract

**Introduction:**

Vacuum extraction is generally considered an operator-dependent task, with most attention directed toward the obstetrician’s technical abilities (1–3). Little is known about the effect of the team and non-technical skills on clinical outcomes in vacuum-assisted delivery. This study aimed to investigate whether the non-technical skills of obstetricians were correlated with their level of clinical performance via the analysis of video recordings of teams conducting actual vacuum extractions.

**Methods:**

We installed between two or three video cameras in each delivery room at Aarhus University Hospital and Horsens Regional Hospital and obtained 60 videos of teams managing vacuum extraction. Appropriate consent was obtained. Two raters carefully reviewed the videos and assessed the teams’ non-technical skills using the Assessment of Obstetric Team Performance (AOTP) checklist, rating all items on a Likert scale score from 1 to 5 (1 = poor; 3 = average; and 5 = excellent). This resulted in a total score ranging from 18 to 90. Two different raters independently assessed the teams’ clinical performance (adherence to clinical guidelines) using the TeamOBS-Vacuum-Assisted Delivery (VAD) checklist, rating each item (0 = not done, 1 = done incorrectly; and 2 = done correctly). This resulted in a total score with the following ranges (low clinical performance: 0–59; average: 60–84; and high: 85–100). Interrater agreement was analyzed using intraclass correlation (ICC), and the risk of high or low clinical performance was analyzed on a logit scale to meet the assumption of normality.

**Results:**

Teams that received excellent non-technical scores had an 81% probability of achieving high clinical performance, whereas this probability was only 12% among teams with average non-technical scores (*p* < 0.001). Teams with a high clinical performance often had excellent behavior in the non-technical items of “team interaction,” “anticipation,” “avoidance fixation,” and “focused communication.” Teams with a low or average clinical performance often neglected to consider analgesia, had delayed abandonment of the attempted vaginal delivery and insufficient use of appropriate fetal monitoring. Interrater reliability was high for both rater-teams, with an ICC for the non-technical skills of 0.83 (95% confidence interval [CI]: 0.71–0.88) and 0.84 for the clinical performance (95% CI: 0.74–0.90).

**Conclusion:**

Although assisted vaginal delivery by vacuum extraction is generally considered to be an operator-dependent task, our findings suggest that teamwork and effective team interaction play crucial roles in achieving high clinical performance. Teamwork helped the consultant anticipate the next step, avoid fixation, ensure adequate analgesia, and maintain thorough fetal monitoring during delivery.

## 1 Introduction

Assisted vaginal delivery is employed in situations where the second stage of labor should be shortened due to maternal or fetal indications. The primary goal is to achieve expedited delivery while maintaining high-quality care. However, delivery wards worldwide have witnessed increased rates of cesarean sections in the second stage of labor. This trend may signify a decline in clinical skills or concerns regarding the risk of severe complications such as maternal perineal trauma, fetal scalp injuries, and even intracranial hemorrhages (1, 2). While vacuum extraction remains the most utilized method for assisted vaginal delivery, we must be vigilant in addressing and mitigating these risks.

Vacuum extraction is commonly perceived as an operator-dependent task, with emphasis placed on the technical skills of the obstetrician (1–3). However, there is a paucity of research exploring the importance of the non-technical skills employed by the entire delivery team (4–6). These non-technical skills have been categorized as cognitive, social, and personal resource skills. They include decision-making, situational awareness, communication, teamwork, leadership, and coping with stress and fatigue (7). These non-technical skills are important in acute emergency teams (8–10) and in obstetric teams managing postpartum hemorrhage (11), settings that clearly differ from delivery by vacuum extraction (12, 13).

In the context of vacuum extraction, all research on the non-technical skills has been confined to simulation settings (4, 5, 14, 15) which may not entirely capture the intricacies of real-word patient care. This underscores the imperative for a thorough evaluation of non-technical skills in the delivery suite (16). Therefore, this study analyzes videos of teams engaged in vacuum extractions during real-life deliveries, aiming to explore the non-technical skills and assess their potential correlation with the level of clinical performance.

## 2 Materials and methods

### 2.1 Study design and setting

Two Danish hospitals participated in the study: Aarhus University Hospital, delivering level 3 maternity care for 5,000 deliveries per year, with 50 physicians, 100 midwives, and 20 technicians; and Horsens Regional Hospital, delivering level 2 maternity care for 2,000 deliveries per year, with 25 physicians, 45 midwives, and 2 technicians. The delivery rooms of the hospitals are well-equipped with two or three high-definition mini-dome surveillance cameras and a ceiling microphone at the center of the room. To minimize the video recordings of normal deliveries, we designed a video system that was activated by Bluetooth on the obstetrician’s phone. All cameras recorded 5-min loops until the obstetrician entered the room; the Bluetooth signal activated the cameras, and the preceding 5 min and subsequent time spent in the delivery room were recorded. Videos were only included if consent was obtained from all participants within 48 h; otherwise, videos were automatically deleted from the server.

Written consent was obtained from all participants appearing in the videos (staff, patients, and relatives). Information and collection of consent was a two-step procedure. First, all women received information about the research project in our outpatient clinic some weeks before labor, with records of this information noted in the medical electronic records. Subsequently, in the delivery ward, the midwife responsible for the delivery ensured written informed consent was obtained. The inclusion criterion encompassed all assisted deliveries conducted in our delivery suites, and the exclusion criteria included the absence of informed consent or technical errors in sound or video recording.

Vacuum extractions in Denmark are mainly conducted in the delivery room, with the opportunity to swiftly move to the theater to perform a cesarean section in cases of failed extraction. The incidence of cesareans is 20%, with approximately half of these being emergencies and half elective. The incidence of attempted vacuum extractions is 6–7% of all deliveries and failed vacuum extractions is approximately 9% of these. The hospitals guideline state that vacuum extraction should be discontinued if no descent occurs after two pulls or two pop-offs, or if delivery is not imminent following three pulls. The vacuum extraction is often conducted by a multidisciplinary team, where the consultant performs the extraction, a midwife supports the perineum, a second midwife ensures fetal monitoring, and a nurse assistant ensures all equipment for the extraction is available (for example, different-sized metal cups or silicone alternatives and vacuum machine). Forceps is rarely used for vertex presentation.

The video recordings of teams were recorded between November 2014 and July 2016 and analyzed during the summer of 2017.

### 2.2 Ethics approval

Ethical and legal approval for this study was obtained in May 2014 from the Central Denmark Region, the Danish Data Protection Agency (2012–58-006), and the Research Foundation of Central Denmark (Record No. 1–16–02-257-14). All videos were included with informed consent in conformity with the Danish code §264.

### 2.3 Clinical performance assessment by team OBS-VAD

We developed and validated a checklist to evaluate the clinical performance of assisted vaginal delivery via vacuum extraction. The checklist was the result of a Delphi process with 12 experts from the United Kingdom, Norway, Iceland, Sweden, and Denmark (17), and comprises 18 items, each with a weight of importance score: “Done correctly and in a timely manner” (2 points); “Done incorrectly or done correctly with delay” (1 point); “Not done” (0 points); “Cannot be assessed” (no value); or “Not indicated” (no value). The checklist results in a total score of 0–100% (100% = highest standard of care). The checklist also included a visual analog scale of patient safety ranging from 0 to 100%, where elements of management not included in the checklist were evaluated. The total clinical performance score was reported as: ‘High’ (85–100); ‘Average’ (60–84); or ‘Low’ (<60). Both raters (LH and LA) were senior obstetric consultants working in one of the two study hospitals. They were experienced in video review and received appropriate training, including an introduction to the TeamOBS-VAD checklist and a discussion of examples of high and low performance. Subsequently, the raters independently assessed all videos for clinical performance, and were blinded to each other’s score.

### 2.4 Non-technical skills assessment by assessment of obstetric team performance

We used the AOTP (18) validated tool to assess the team’s non-technical score. It comprises 18 items grouped into six categories: “Communication with the patient,” “Task management,” “Teamwork,” “Situation awareness,” “Communication with the team members,” and “Environment of the room.” Each item was scored on a Likert scale (1–5; 1, poor; 5, excellent), resulting in a total score between 18 and 90 (list of all items in Supplementary Table S3). Both raters (LB and KH) were physicians who were experienced in video review and AOTP from a previous study (11). The raters assessed all the videos independently and were blinded to each other’s scores.

### 2.5 Statistical analysis

We assessed the following criteria: (1) the interrater agreement by intraclass correlation (ICC) and Bland–Altman analysis; (2) the agreement on each item by percentage agreement and weighed Cohen’s kappa; (3) the association between the non-technical score and clinical performance by a restricted cubic spline regression analysis with three knots at 2.5, 3, and 3.5; and (4) the mean difference in clinical performance between the lowest (2) and the highest non-technical score (5) by spline regression analysis (19, 20). The regression models were checked using the diagnostic plots of the residuals. The confidence intervals for risk analysis were computed using a non-parametric percentile bootstrap. The potential confounding factors of parity, indication for vacuum (prolonged second stage or fetal compromise), classification (mid, low, and outlet), level of training (junior 1–5 years and senior >5 years), time of event, and hospital type were assessed using multiple linear regression analysis. We used STATA 15 (StataCorp LP, College Station, TX) for all statistical analysis.

## 3 Results

### 3.1 Included videos

**Figure 1:**
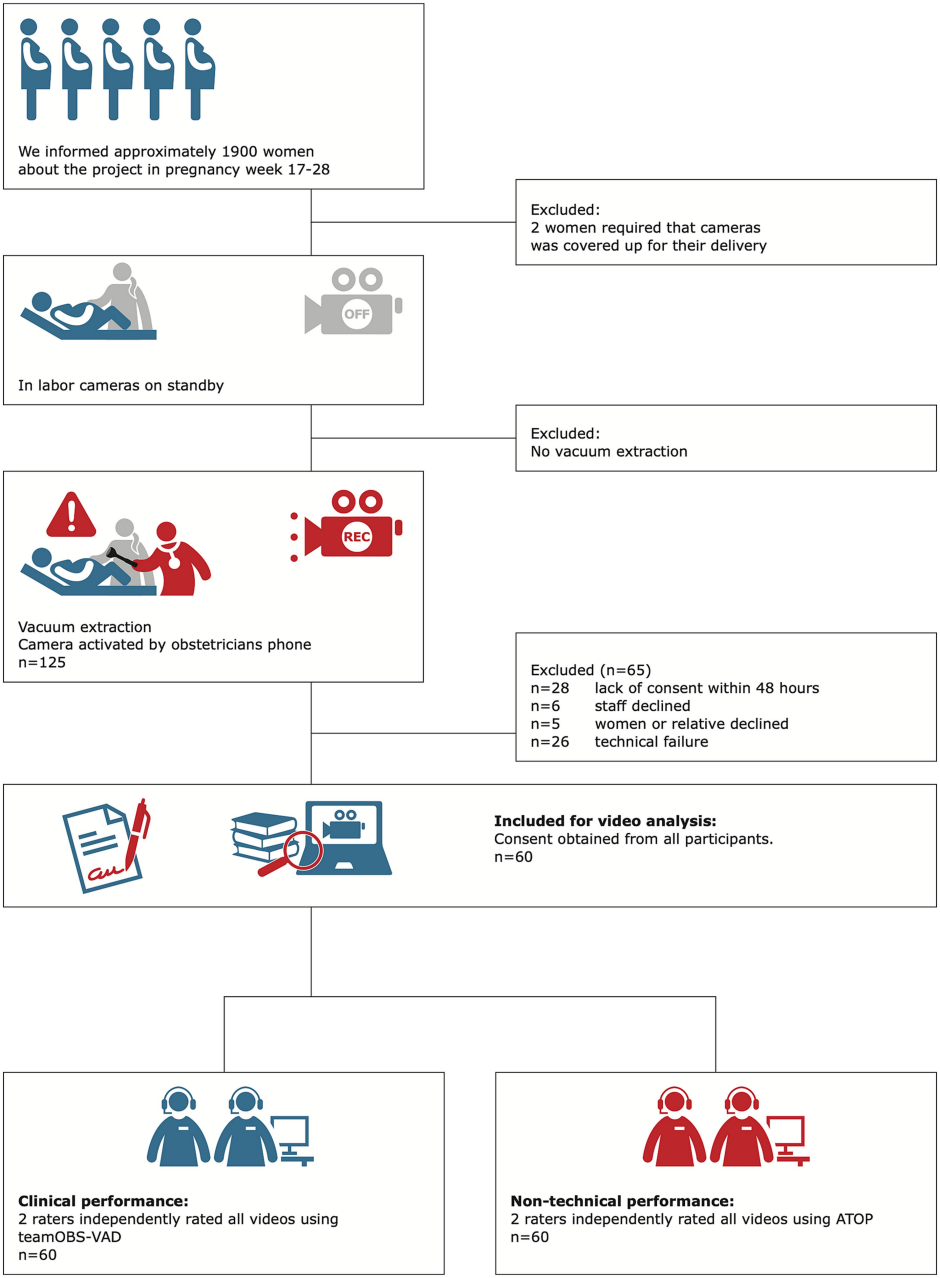
Inclusion of teams.

Women expecting to give birth in one of the two study hospitals were informed about the project, and videos between November 2014 and July 2016 were included. The main reason for exclusion was the absence of consent after 48 h (28 cases), often because the mother had been discharged from the hospital before consent was obtained. Consent was declined by five healthcare providers, six mothers, or relatives. We included 60 videos, with 48% eligible cases (Figure 1). The 60 teams comprised 178 different healthcare providers, 60 different team combinations, and different levels of difficulty with vaginal delivery (Table 1).

**Table 1:** Description of included teams.

		n	Mean/median/proportion
Team size, mean (SD)		60	4.5 (0.9)
Women previous (prior) vaginal delivery			
	1. No, n (%)	39	65%
	2. Yes, n (%)	5	8%
	3. Information not verbalized in video, n (%)	16	27%
Indication of vacuum extraction			
	1. Prolonged second stage of labor or for maternal benefit, n (%)	30	50%
	2. Suspicion of immediate or potential fetal compromise, n (%)	30	50%
Classification of assisted vaginal delivery*			
	1. Mid, n (%)	15	25%
	2. Low, n (%)	20	33%
	3. Outlet, n (%)	11	19%
	4. Information not verbalized in video, n (%)	14	23%
Choice of vacuum extractor			
	1. Soft vacuum cup, n (%)	15	25%
	2. Metal vacuum cup, n (%)	39	65%
	3. Replacing soft cup to metal, n (%)	6	10%
Procedure of the vacuum extraction			
	1. Delivery conducted in ≤3 contractions	48	80%
	2. Delivery conducted in >3 contractions	12	20%
	3. Delivery with cup detachment, n (%)	10	17%
	4. Duration of vacuum applied (min), median (IQR)	60	4.5 (2–9)
	5. Failed vacuum extraction, n (%)	4	6%
Experience of operator/ level of training			
	1. Resident, registrar, 1–5 years’ experience, n (%)	25	42%
	2. Consultant, >5 years’ experience, n (%)	35	58%
Time of event			
	1. Day (7:00–14:59), n (%)	18	30%
	2. Evening (15:00–23:59), n (%)	23	38%
	3. Night (00:00–06:59), n (%)	19	32%
Hospital type			
	1. University hospital, maternal care level 3, n (%)	32	53%
	2. Regional hospital, maternal care level 2, n (%)	28	47%

*The American College of Obstetricians and Gynecologist classification of types of operative vaginal delivery (ref guideline ACOG).

### 3.2 Interrater agreement

The interrater agreement was high, as raters assessing the non-technical skills had an ICC of 0.83 (95% CI: 0.71–0.88) and consultants assessing clinical performance had an ICC of 0.84 (95% CI: 0.74–0.90). Agreement among raters for assessing the specific non-technical skills was 0.69–0.94 weighted kappa and agreement was visualized using Bland–Altman Plots and limits of agreement (Table 2; Supplementary Tables S1, S2). Four videos were discussed and reassessed by the two raters as their clinical performance assessments differed by >15%, which had been defined a priority as the maximum acceptable difference. This new consensus score was used in the risk analyses, but not in the ICC calculations. No videos were reassessed for non-technical scores.

**Table 2:** Interrater agreement for clinical performance and non-technical performance.

	Descriptive		ICC (95% CI)			
	Mean	Range	Individual rater*		Average of two raters**	
Clinical performance: TeamOBS-VAD score	83.2	(49–100)	0.73	(0.58–0.82)	0.84	(0.74–0.90)
Non-technical performance: ATOP average score	70.5	(39–87)	0.71	(0.55–0.82)	0.83	(0.71–0.88)

*Intraclass correlation (ICC) between raters. **All 60 videos were analyzed by two raters. The average agreement represents the ICC between two raters and the other two raters.

### 3.3 Clinical performance and non-technical skills

Most teams achieved high clinical performance scores in ensuring the correct position of the mother (94%), appropriate number of staff present (91%), and delivery conducted within maximum four pulls (88%). The greatest challenge posed in clinical skill was considering analgesia, as 39% of the teams neither discussed nor mentioned analgesia as an option.

**Figure 2:**
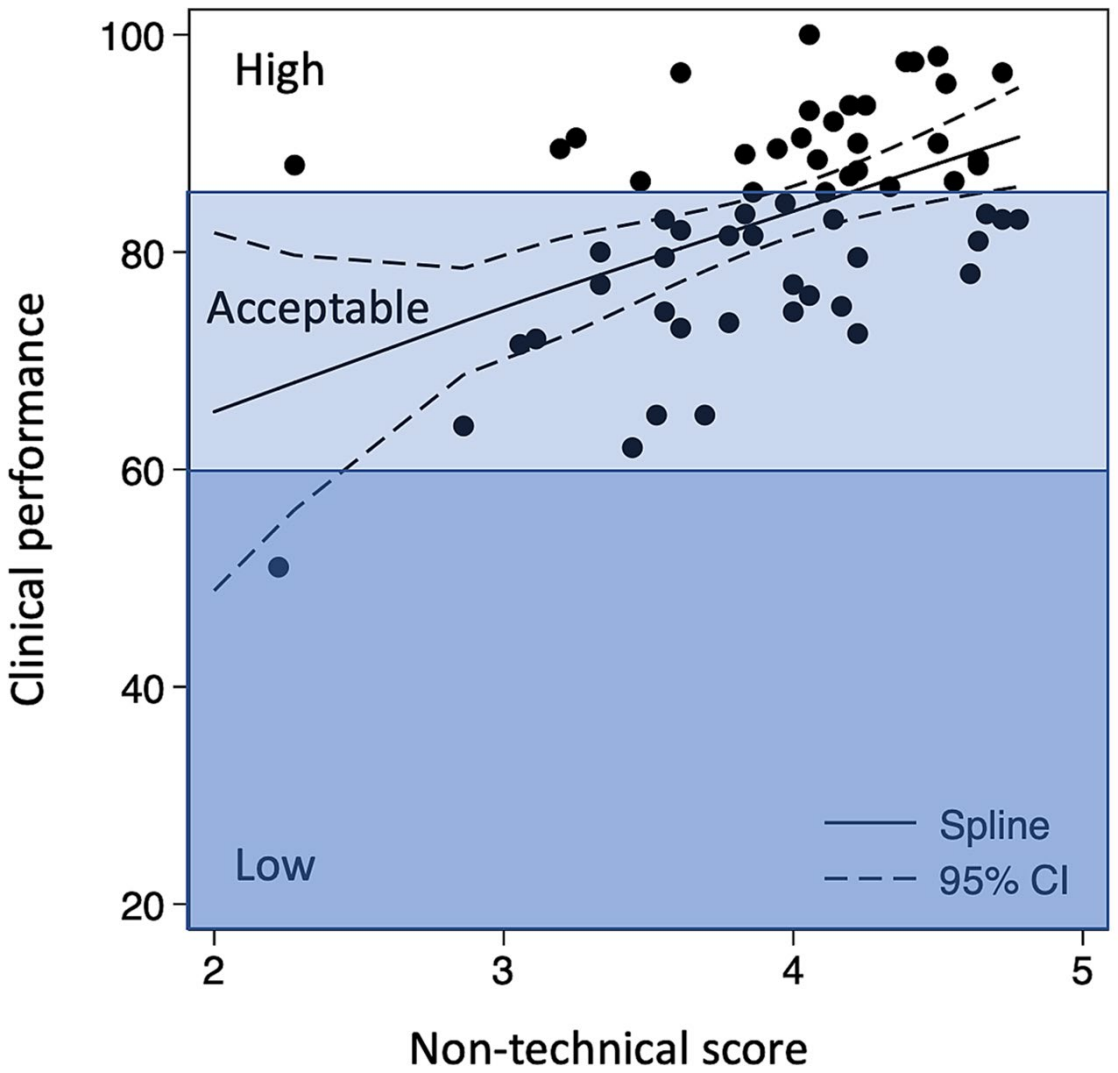
Clinical performance and non-technical skills.

Clinical performance and non-technical skills correlated with a ‘dose–response-like’ association (Figure 2). Teams with excellent non-technical scores had an 81% probability of high clinical performance, whereas this probability was only 12% among teams with average non-technical scores (*p* < 0.001; Table 3). Clinically high-performing teams demonstrated excellent behavior in the non-technical skills categories of team interaction, anticipation, avoidance of fixation, and focused communication (Supplementary Table S3). These results were robust in terms of the following confounders: hospital, team size, time of day, and number of pulls during the delivery (Supplementary Table S4).

**Table 3:** Risk/chance of low/high clinical performance to the level of non-technical performance.

Non-technical score	Clinical performance: risk of low score*		Clinical performance: chance of high score**	
3 (Average)	4.3%	(0.2–12.4%)	12.3%	(2.0–29.2%)
4 (Good)	0.3%	(0.0–0.9%)	43%	(34.5–54.0%)
5 (Excellent)	0.01%	(≤0.01–0.01%)	80.6%	(63.7–94.1%)

These figures are based on 60 recordings from real-life teams managing major vaginal-assisted deliveries via vacuum extraction and risk (95% CI). *TeamOBS-VAD score below the minimum pass (score < 60). **TeamOBS-VAD score above high performance (score > 85).

## 4 Discussion

Assisted vaginal delivery by vacuum extraction has been considered an operator-dependent task; however, our findings indicate that the non-technical skills of teams play an important role in achieving high clinical performance. Thus, teams with an excellent non-technical score had an 81% chance of achieving high clinical performance, whereas this probability was only 12% among teams with average non-technical scores. Teamwork helped the obstetrician to anticipate the next steps, avoid fixation, ensure analgesia, and ensure sufficient fetal monitoring during delivery.

The main strength of this study is the automatic inclusion of videos using Bluetooth in the obstetrician’s telephone, which activated the video cameras whenever the obstetrician entered the delivery room (21). This potentially reduced selection bias. The sample size is another strength, as the sample included 60 different teams and 178 healthcare providers from two hospitals, operators of the vacuum at all levels of experience, all degrees of difficulty, every day of the week, and any time of day. Finally, the two pairs of raters used a validated checklist for systematic assessment and the interrater agreement was high.

Our study had certain limitations. We cannot exclude selection bias, as low-performing teams may have been less willing to provide their consent. However, it is reassuring that 95% of the healthcare providers gave consent to include their videos and that only two videos were deleted after staff withdrew their consent. We also acknowledge that our setup did not include all vacuum extractions, as the camera was activated by the consultant on call. There is a risk of assessment bias as AOTP raters may have been influenced by the actual clinical performance and vice versa. All teams were aware of the project, and this may have changed their behavior, a form of “Hawthorne effect” which might affect the observed association (22). Furthermore, despite having all the delivery rooms equipped with two or three cameras in the ceiling, the video could not capture all the details of the technical skills, and raters often missed tactile information gained during vaginal examination before the operator applied the cup (for example, molding, level, and position). Furthermore, our legal permission was restricted to analyze the videos; hence, we did not have access to any medical journal with maternal or neonatal outcomes. Finally, while our study found that clinical performance and non-technical scores were strongly correlated with a dose–response like association; this is no proof of causality.

The opportunity to review video recordings of teams managing vacuum extractions in real-life was a special experience that changed the way vacuum extractions were handled in our department (23). Epidural as pain relief in labor is available for every laboring woman in Denmark; however, uptake is driven by maternal request. Overall, approximately 27% of nulliparous women in labor request an epidural. We considered whether the high sound level on the recording reflected real life. Therefore, we tested our audio source with the help of a sound engineer and found that the quality was high and that the dB level in the room was reflected by the dB level in the video (24). These findings highlight the importance of improving our practice of offering women analgesia during vacuum extraction. Based on our study, we recommend prioritizing the wider use of pudendal block in cases of vacuum extraction and ensuring that women are adequately informed about assisted delivery preferable in the antenatal period (25).

Before this study, non-technical skills for vacuum extraction have only been investigated in simulated settings (6, 15, 26–28), but did suggest that non-technical skills contribute to successful delivery assisted by vacuum extraction (4, 14, 29). However, these studies did not describe the specific aspects of behavior or the specific non-technical skills that support teams to achieve high clinical performance. In this study, we observed how enacting specific non-technical skills helped obstetricians to anticipate the next steps, such as offering analgesia to women without an epidural or calling the pediatric team to the room when the indication was suspected fetal distress. Moreover, the team helped the consultant to keep track of the time and the number of contractions, thereby helping the consultant decide when to abandon the attempted vaginal delivery in a timely manner. This calls for rethinking our learning path for vacuum extraction, ensuring that trainees learn to include effective teamwork behavior in training and practice. Thus, this study confirms the results on surgical (30), trauma (31, 32), and resuscitation teams (33–35) regarding the importance of non-technical skills in providing high clinical performance.

High-performing teams in this study used specific behaviors that differed from those of high-performing teams managing postpartum hemorrhage (11). High-performing teams managing vacuum extraction demonstrated high scores in team interaction, anticipation, avoidance of fixation, and focused communication, whereas high-performing teams managing postpartum hemorrhage had high scores in vigilance, role assignment, stress management, and leadership (11). This difference may reflect the fact that most vacuum extractions are a ‘semi-elective’ procedure, whereas postpartum hemorrhage is usually an urgent, acute emergency. Another interesting disparity between the two settings was the importance of avoiding fixation, which we could only demonstrate in the vacuum extraction setting but not in the postpartum hemorrhage setting. This is related to the fact that the accoucheur who is conducting the vacuum delivery is ‘task-focused’ and therefore cannot maintain wider situational awareness. This requires a member of the team to ‘step back’ to monitor the whole situation (that is, ‘situational leadership’). This tends to happen in teams managing acute emergencies, such as postpartum hemorrhage, where the situational leader steps back to co-ordinate and delegate tasks while ensuring that vigilance is maintained (7). Therefore, all team members should keep an eye on the overall situation while the accoucheur conducts the vacuum extraction and may need to consider whether an individual needs to ‘step back’ to maintain overall vigilance.

Video reviews offer a unique opportunity to review our performance, and future studies should investigate whether video reviews can help to improve our vacuum extraction performance (31, 34). Obstetric trainees primarily learn to perform vacuum extraction from senior colleagues by craft apprenticeship. In the last few decades, trainees have experienced difficulties in developing and maintaining obstetrical competencies, such as vacuum extractions, due to reduced working hours and a reduced rate of instrumental deliveries in obstetric care (33). Elective video review could be a valuable learning method to junior obstetricians to improve performance as they can revisit and reflect on their performance and learn from experienced colleagues (36). When they become consultants, they primarily work alone or with a junior trainee, and do not always have the same opportunity to benchmark their performance with several consultants; feedback may not always be ideal. Similar to other studies, we found that our colleagues had a genuine interest in learning about the outcomes of the video review, and we think video reviews offer us new learning opportunities for vacuum extraction to improve and maintain high performance in obstetric teams. Future studies are needed to explore how (37).

The external validity of our findings must be considered before they can be applied in other settings. In the labor and delivery wards, where the standard analgesia is epidural/spinal, our findings on further analgesia may not be relevant. However, we believe that the key findings of this study may be applicable in other settings where teams manage vacuum extractions, and we recommend including the specific behaviors of team interaction, anticipation, avoiding fixation, and focused communication to complement any technical training on vacuum extraction.

In conclusion, mastering vacuum extraction is a core competency in delivery wards and is important for the safety of women and children. Our study demonstrated how video reviews of real-life vacuum extraction can be used to evaluate the clinical and non-technical performance of teams to identify areas that require particular attention during training. Although vaginal vacuum extraction is generally accepted as an operator-dependent task, our findings indicate that the teams’ non-technical skills play an important role in ensuring high clinical performance, as the accoucheur who is conducting the vacuum delivery is “task-focused” and cannot maintain wider situational awareness. Therefore, the team members are required to “step back” to monitor the whole situation, and the key non-technical skills associated with high clinical performance were team interaction, anticipation, avoidance of fixation, and focused communication.

## Data availability statement

The raw data supporting the conclusions of this article will be made available by the authors, without undue reservation.

## Ethics statement

The studies involving humans were approved by Ethical and legal approval for this study was obtained in May 2014 from the Central Denmark Region, the Danish Data Protection Agency (2012-58-006), and the Research Foundation of Central Denmark (Record No. 1-16-02-257-14). All videos were included with informed consent in conformity with the Danish code §264. The studies were conducted in accordance with the local legislation and institutional requirements. The participants provided their written informed consent to participate in this study.

## Author contributions

LB: Conceptualization, Data curation, Formal analysis, Funding acquisition, Investigation, Methodology, Project administration, Resources, Software, Validation, Visualization, Writing – original draft. LR: Investigation, Writing – review & editing. KH-H: Data curation, Formal analysis, Investigation, Methodology, Writing – review & editing. LH: Conceptualization, Data curation, Formal analysis, Investigation, Methodology, Supervision, Writing – review & editing. KH: Conceptualization, Methodology, Writing – review & editing. OK: Conceptualization, Funding acquisition, Investigation, Methodology, Project administration, Resources, Supervision, Writing – review & editing. NU: Conceptualization, Formal analysis, Investigation, Methodology, Project administration, Supervision, Validation, Writing – review & editing. TM: Conceptualization, Formal analysis, Investigation, Methodology, Supervision, Validation, Visualization, Writing – review & editing.

## References

[1] Vacca A. Vacuum-assisted delivery: an analysis of traction force and maternal and neonatal outcomes. Aust N Z J Obstet Gynaecol. (2006) 46:124–7. doi: 10.1111/j.1479-828X.2006.00540.x, PMID: 16638034

[2] Sau A Sau M Ahmed H Brown R. Vacuum extraction: is there any need to improve the current training in the UK? Acta Obstet Gynecol Scand. (2004) 83:466–70. doi: 10.1111/j.0001-6349.2004.0399.x, PMID: 15059160

[3] Bahl R Murphy DJ Strachan B. Qualitative analysis by interviews and video recordings to establish the components of a skilled rotational forceps delivery. Eur J Obstet Gynecol Reprod Biol. (2013) 170:341–7. doi: 10.1016/j.ejogrb.2013.06.034, PMID: 23891388

[4] Bahl R Murphy DJ Strachan B. Non-technical skills for obstetricians conducting forceps and vacuum deliveries: qualitative analysis by interviews and video recordings. Eur J Obstet Gynecol Reprod Biol. (2010) 150:147–51. doi: 10.1016/j.ejogrb.2010.03.004, PMID: 20362383

[5] Bracco F Masini M De Tonetti G Brogioni F Amidani A Monichino S. Adaptation of non-technical skills behavioural markers for delivery room simulation. BMC Pregnancy Childbirth. (2017) 17:89. doi: 10.1186/s12884-017-1274-z, PMID: 28302085 PMC5356378

[6] Chang YS Coxon K Portela AG Furuta M Bick D. Interventions to support effective communication between maternity care staff and women in labour: a mixed-methods systematic review. Midwifery. (2018) 59:4–16. doi: 10.1016/j.midw.2017.12.014, PMID: 29351865 PMC5852259

[7] Flin R O’Connor P Crichton M. Safety at the sharp end: A guide to non-technical skills; chapter 2. US: CRC Press. (2008);17–40.

[8] Joint Commission on Accreditation of Healthcare Organizations, USA.. Preventing maternal death. Sentinel Event Alert. (2010) 26:1–4. 20183946

[9] Guise JM Segel S. Teamwork in obstetric critical care. Best Pract Res Clin Obstet Gynaecol. (2008) 22:937–51. doi: 10.1016/j.bpobgyn.2008.06.010, PMID: 18701352 PMC4987289

[10] Andel C Davidow SL Hollander M Moreno DA. The economics of health care quality and medical errors how big a problem is quality and patient safety? J Health Care Finance. (2012) 39:39–50. PMID: 23155743

[11] Brogaard L Kierkegaard O Hvidman L Jensen KR Musaeus P Uldbjerg N. The importance of non-technical performance for teams managing postpartum haemorrhage: video review of 99 obstetric teams. BJOG. (2019) 126:1015–23. doi: 10.1111/1471-0528.15655, PMID: 30771263

[12] Leonard M Graham S Bonacum D. The human factor: the critical importance of effective teamwork and communication in providing safe care. Qual Saf Health Care. (2004) 13:i85–90. doi: 10.1136/qshc.2004.010033 15465961 PMC1765783

[13] Patey R Flin R Flwtcher G Nicola Mara RG. Developing a taxonomy of anesthetists’ nontechnical skills (ANTS). Advan Patient Safety. (2005) 4:1–12. 21250033

[14] Bahl R Murphy DJ Strachan B. Decision-making in operative vaginal delivery: when to intervene, where to deliver and which instrument to use? Qualitative analysis of expert clinical practice. Eur J Obstet Gynecol Reprod Biol. (2013) 170:333–40. doi: 10.1016/j.ejogrb.2013.06.033, PMID: 23910696

[15] Gruise JJ. HHS Public Access. (2008) 34:352–9.

[16] Hinshaw K. Non-technical skills to improve obstetric practice In: Arulkumaran SS, editor. Best practice in labour and delivery [internet]. 2nd ed. Cambridge: Cambridge University Press (2016). 389–400.

[17] Brogaard L Hinshaw K Kierkegaard O Manser T Uldbjerg N Hvidman L. Developing the TeamOBS-vacuum-assisted delivery checklist to assess clinical performance in a vacuum-assisted delivery: a Delphi study with initial validation. Front Med (Lausanne). (2024) 11:1330443. doi: 10.3389/fmed.2024.1330443 38371513 PMC10869485

[18] Morgan PJ Tregunno D Pittini R Tarshis J Regehr G Desousa S. Determination of the psychometric properties of a behavioural marking system for obstetrical team training using high-fidelity simulation. BMJ Qual Saf. (2012) 21:78–82. doi: 10.1136/bmjqs-2011-000296, PMID: 21994358

[19] Carstensen B. Comparing clinical measurement methods: A practical guide. US: John Wiley & Sons, Ltd. (2010).

[20] Bland JM Altman DG. Applying the right statistics: analyses of measurement studies Ultrasound Obstet Gynecol. (2003) 22:85–93. doi: 10.1002/uog.122, 12858311

[21] Brogaard L Uldbjerg N. Filming for auditing of real-life emergency teams: a systematic review. BMJ Open Qual. (2019) 8:e000588. doi: 10.1136/bmjoq-2018-000588, PMID: 31909207 PMC6937091

[22] McCarney R Warner J Iliffe S van Haselen R Griffin M Fisher P. The Hawthorne effect: a randomised, controlled trial. BMC Med Res Methodol. (2007) 7:30. doi: 10.1186/1471-2288-7-30, PMID: 17608932 PMC1936999

[23] RKKP. 2022 [cited 2023 Oct 18]. Dansk kvalitetsdatabase for fødsler. (2022). Available from: https://www.sundhed.dk/content/cms/66/4666_dkf-aarsrapport-2022_offentlig.pdf

[24] Jensen KR Hvidman L Kierkegaard O Gliese H Manser T Uldbjerg N. Noise as a risk factor in the delivery room: a clinical study. PloS One. (2019) 14:e0221860. doi: 10.1371/journal.pone.0221860, PMID: 31469866 PMC6716652

[25] Murphy DJ Strachan BK Bahl R. Assisted vaginal birth: green-top guideline no. 26. BJOG. (2020) 127:e70–e112. doi: 10.1111/1471-0528.16092, PMID: 32346983

[26] Siassakos D Bristowe K Draycott TJ Angouri J Hambly H Winter C. Clinical efficiency in a simulated emergency and relationship to team behaviours: a multisite cross-sectional study. BJOG. (2011) 118:596–607. doi: 10.1111/j.1471-0528.2010.02843.x 21291509

[27] Cornthwaite K Edwards S Siassakos D. Reducing risk in maternity by optimising teamwork and leadership: an evidence-based approach to save mothers and babies. Best Pract Res Clin Obstet Gynaecol. (2013) 27:571–81. doi: 10.1016/j.bpobgyn.2013.04.004, PMID: 23647702

[28] Siassakos D Draycott T Montague I Harris M. Content analysis of team communication in an obstetric emergency scenario. J Obstet Gynaecol (Lahore). (2009) 29:499–503. doi: 10.1080/01443610903039153, PMID: 19697196

[29] Edozien LC. Towards safe practice in instrumental vaginal delivery. Best Pract Res Clin Obstet Gynaecol. (2007) 21:639–55. doi: 10.1016/j.bpobgyn.2007.03.006, PMID: 17466598

[30] Parker SH Flin R McKinley A Yule S. Factors influencing surgeons’ intraoperative leadership: video analysis of unanticipated events in the operating room. World J Surg. (2014) 38:4–10. doi: 10.1007/s00268-013-2241-0, PMID: 24114366

[31] Hoyt DB Shackford SR Fridland PH Mackersie RC Hansbrough JF Wachtel TL. Video recording trauma resuscitations: an effective teaching technique. J Trauma. (1988) 28:435–40. doi: 10.1097/00005373-198804000-00003, PMID: 3352005

[32] Townsend RN Clark R Ramenofsky ML Diamond DL. ATLS-based videotape trauma resuscitation review: education and outcome. J Trauma. (1993) 34:133–8. doi: 10.1097/00005373-199301000-00025, PMID: 7679741

[33] Finer NN Rich W. Neonatal resuscitation: toward improved performance. Resuscitation. (2002) 53:47–51. doi: 10.1016/S0300-9572(01)00494-4, PMID: 11947979

[34] Jiang C Zhao Y Chen Z Chen S Yang X. Improving cardiopulmonary resuscitation in the emergency department by real-time video recording and regular feedback learning. Resuscitation. (2010) 81:1664–9. doi: 10.1016/j.resuscitation.2010.06.023, PMID: 20727657

[35] Brogaard L Hvidman L Esberg G Finer N Hjorth-Hansen KR Manser T. Teamwork and adherence to guideline on newborn resuscitation-video review of neonatal interdisciplinary teams. Front Pediatr. (2022) 10:828297. doi: 10.3389/fped.2022.828297, PMID: 35265565 PMC8900704

[36] Lesch H Johnson E Peters J Cendán JC. VR simulation leads to enhanced procedural confidence for surgical trainees. J Surg Educ. (2020) 77:213–8. doi: 10.1016/j.jsurg.2019.08.008, PMID: 31466895 PMC8041454

[37] Davis L Johnson L Allen SR Kim PK Sims CA Pascual JL. Practitioner perceptions of trauma video review. J Trauma Nurs. (2013) 20:150–4. doi: 10.1097/JTN.0b013e3182a172b6, PMID: 24005118

