# Supplementary material for: Team performance during vacuum-assisted vaginal delivery: video review of obstetric multidisciplinary teams
Source: Front Med (Lausanne). 2024 Mar 20;11:1330457. doi: 10.3389/fmed.2024.1330457 (PMC10987771; doi:10.3389/fmed.2024.1330457)
Supplement: Supplementary file 1 [file Data_Sheet_1.docx]

Supplementary Material


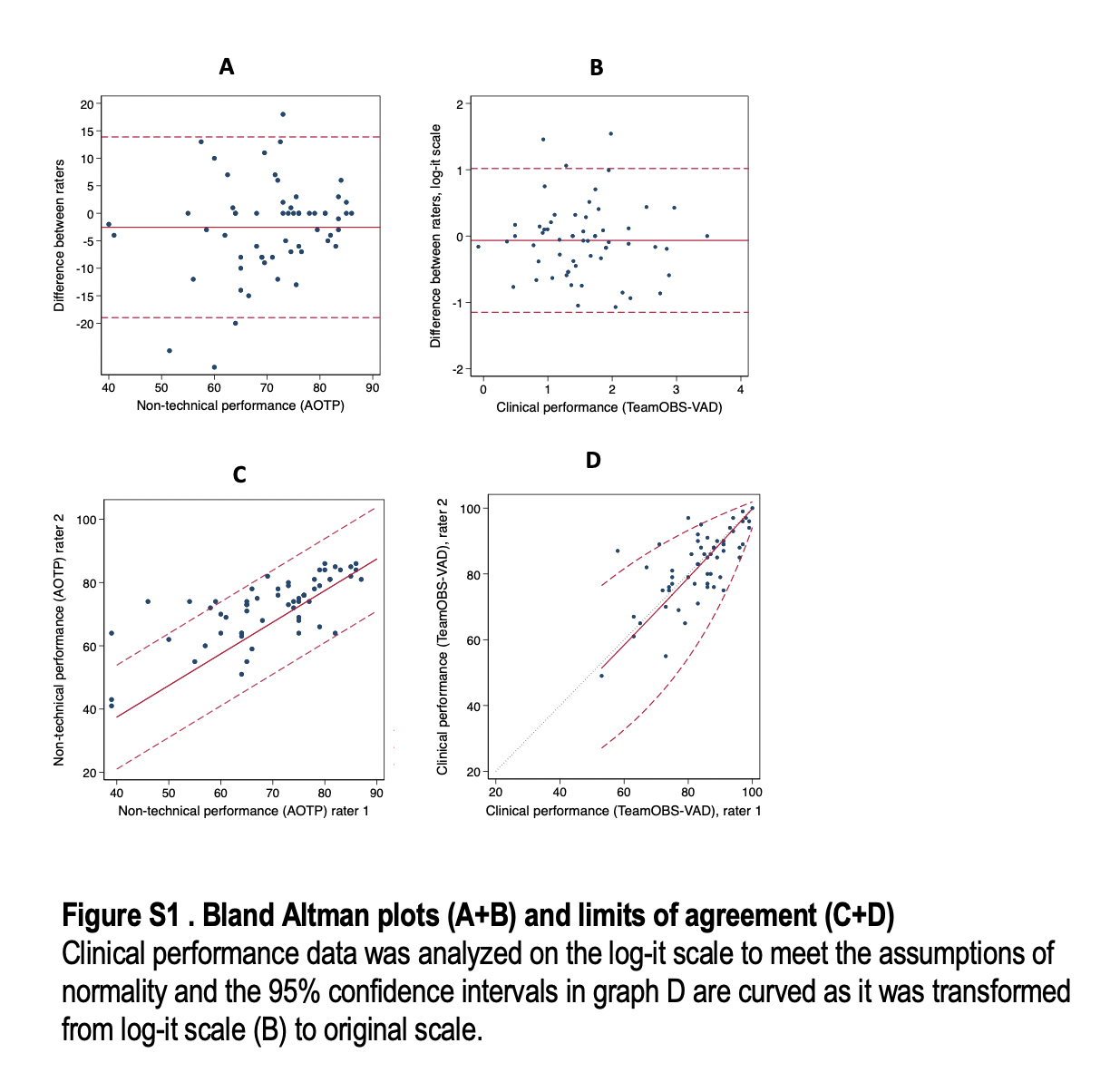


**Figure S1. Bland–Altman plots (A+B) and limits of agreement (C+D).** Clinical performance data was analyzed on the log-it scale to meet the assumptions of normality. The 95% confidence intervals in (D) are curved, as this was transformed from a log-it scale (B) to an original scale.

**Table S2.** Inter-rater agreement for non-technical performance on the item level

|  | Descriptive | | Kappa | Difference (%) * | | | | |
| --- | --- | --- | --- | --- | --- | --- | --- | --- |
| Items | Mean | Range | Weighted | diff=0 | diff=1 | diff=2 | diff=3 | diff=4 |
| Item 1.1 | 4.1 | (2–5) | 0.82 | 0.58 | 0.32 | 0.1 | 0.00 | 0.00 |
| Item 1.2 | 3.9 | (1–5) | 0.85 | 0.58 | 0.28 | 0.83 | 0.05 | 0.00 |
| Item 1.3 | 4.0 | (1–5) | 0.85 | 0.53 | 0.37 | 0.08 | 0.00 | 0.02 |
| Item 2.1 | 3.7 | (1–5) | 0.87 | 0.58 | 0.32 | 0.08 | 0.02 | 0.00 |
| Item 2.2 | 4.6 | (1–5) | 0.94 | 0.83 | 0.12 | 0.02 | 0.03 | 0.01 |
| Item 2.3 | 4.8 | (3–5) | 0.81 | 0.59 | 0.54 | 0.32 | 0.00 | 0.00 |
| Item 3.1 | 3.6 | (1–5) | 0.83 | 0.52 | 0.35 | 0.08 | 0.05 | 0.00 |
| Item 3.2 | 3.8 | (1–5) | 0.88 | 0.58 | 0.37 | 0.05 | 0.00 | 0.00 |
| Item 3.3 | 4.2 | (1–5) | 0.83 | 0.55 | 0.30 | 0.10 | 0.03 | 0.02 |
| Item 4.1 | 3.7 | (1–5) | 0.88 | 0.65 | 0.23 | 0.10 | 0.01 | 0.01 |
| Item 4.2 | 4.1 | (1–5) | 0.69 | 0.73 | 0.18 | 0.08 | 0.00 | 0.00 |
| Item 4.3 | 4.2 | (1–5) | 0.86 | 0.58 | 0.27 | 0.13 | 0.02 | 0.00 |
| Item 4.4 | 4.4 | (1–5) | 0.85 | 0.58 | 0.30 | 0.08 | 0.02 | 0.02 |
| Item 4.5 | 4.2 | (1–5) | 0.82 | 0.58 | 0.35 | 0.02 | 0.05 | 0.00 |
| Item 5.1 | 3.6 | (2–5) | 0.88 | 0.65 | 0.35 | 0.00 | 0.00 | 0.00 |
| Item 5.2 | 1.2 | (1–3) | 0.94 | 0.90 | 0.08 | 0.02 | 0.00 | 0.01 |
| Item 6.1 | 4.5 | (3–5) | 0.76 | 0.60 | 0.32 | 0.08 | 0.00 | 0.00 |
| Item 6.2 | 3.7 | (1–5) | 0.84 | 0.58 | 0.25 | 0.13 | 0.03 | 0.00 |

*****Difference in percentage. For example, the raters agreed perfectly with a difference of 0 on item 1.1 in 58% of the 61 teams.

**Table S3:** Specific behaviors linked to high clinical performance, analyzed by mean difference - unadjusted

|  | Clinical Performance (95% CI) | | | | | | |
| --- | --- | --- | --- | --- | --- | --- | --- |
| Category/Item | Non-technical score: 2 (poor) | | Non-technical score: 5 (excellent) | | Mean Difference | | Unadjusted *p-v*alue |
| Non-technical score* | 65.3 | (48.8 to 81.8) | 92.5 | (87.0 to 98.1) | 27.2 | (10.5 to 43.9) | 0.002 |
| **1. Communication with patient** | **76.4** | **(66.1 to 86.6)** | **85.7** | **(81.4 to 90.1)** | **9.4** | **(-1.3 to 20.1)** | **0.084** |
| 1.1 Information sharing | 74.0 | (62.6 to 85.4) | 84.2 | (80.5 to 87.8) | 10.1 | (-1.6 to 21.8) | 0.088 |
| 1.2 Reassuring attitude | 78.8 | (73.9 to 83.7) | 87.1 | (83.1 to 91.1) | 8.3 | (1.7 to 14.9) | 0.015 |
| 1.3 Partner management | 82.6 | (74.6 to 90.7) | 85.8 | (81.5 to 90.1) | 3.1 | (-5.9 to 12.2) | 0.489 |
| **2. Task/Case management** | **54.9** | **(39.6 to 70.2)** | **85.7** | **(82.5 to 88.9)** | **30.8** | **(15.1 to 46.5)** | **0.000** |
| 2.1 Plan of action | 74.1 | (68.1 to 80.1) | 85.3 | (81.9 to 88.8) | 11.2 | (4.3 to 18.1) | 0.002 |
| 2.2 Resources utilization | 72.3 | (63.7 to 80.8) | 83.9 | (81.2 to 86.6) | 11.6 | (2.5 to 20.8) | 0.013 |
| 2.3 Problem solving | NA | NA | 85.4 | (82.6 to 88.1) | NA | NA | 0.003 |
| **3. Teamwork** | **70.1** | **(58.9 to 81.4)** | **87.9** | **(82.9 to 92.8)** | **17.7** | **(6.1 to 29.4)** | **0.004** |
| 3.1 Leadership | 77.1 | (71.9 to 82.4) | 86.7 | (81.9 to 91.4) | 9.6 | (2.4 to 16.7) | 0.009 |
| 3.2 Role assignment | 80.2 | (73.8 to 86.6) | 89.3 | (84.4 to 94.1) | 9.1 | (0.7 to 17.4) | 0.033 |
| 3.3 Team interaction | 61.9 | (50.7 to 73.1) | 85.3 | (81.9 to 88.7) | 23.4 | (11.7 to 35.1) | 0.000 |
| **4. Situation awareness** | **70.4** | **(61.4 to 79.4)** | **88.1** | **(84.5 to 91.7)** | **17.7** | **(7.9 to 27.5)** | **0.001** |
| 4.1 Anticipation | 70.5 | (63.3 to 77.6) | 87.0 | (83.9 to 90.1) | 16.5 | (8.6 to 24.4) | 0.000 |
| 4.2 Realizing limitations | 80.1 | (76.0 to 84.1) | 85.0 | (81.3 to 88.7) | 4.9 | (-0.9 to 10.7) | 0.097 |
| 4.3 Avoiding fixation | 73.3 | (66.1 to 80.5) | 88.0 | (84.4 to 91.6) | 14.7 | (6.3 to 23.1) | 0.001 |
| 4.4 Responsiveness | 71.8 | (63.9 to 79.7) | 86.1 | (82.8 to 89.4) | 14.3 | (5.3 to 23.3) | 0.002 |
| 4.5 Vigilance | 78.8 | (65.3 to 92.3) | 88.4 | (85.1 to 91.7) | 9.5 | (-4.1 to 23.2) | 0.167 |
| **5. Team communication** | **79.4** | **(76.1 to 82.7)** | **NA** | **NA** | **NA** | **NA** | **NA** |
| 5.1 Focused communication | 76.3 | (62.6 to 89.9) | 91.6 | (85.2 to 97.9) | 15.3 | (1.3 to 29.3) | 0.033 |
| 5.2 Closing the loop | 88.3 | (80.4 to 96.2) | NA | NA | NA | NA | NA |
| **6. Environment of the room** | **83.5** | **(67.7 to 99.3)** | **88.2** | **(84.5 to 92.0)** | **4.7** | **(-11.2 to 20.7)** | **0.554** |
| 6.1 Management of disruption | NA | NA | 85.3 | (82.0 to 88.6) | NA | NA | NA |
| 6.2 Atmosphere in the room | 76.1 | (71.0 to 81.1) | 87.3 | (83.4 to 91.3) | 11.3 | (4.7 to 17.8) | 0.001 |

Data represents the scores from 60 teams managing vacuum extraction

*Represents the average ATOP score = (sum of 18 items score/18)
NA, non-applicable due to the distribution of data

**Table S4:** Specific behaviors linked to high clinical performance, analyzed by mean difference - adjusted

|  | **Unadjusted** | | | **Adjusted for hospital** | | | | **Adjusted for pulls** | | | | **Adjusted for indication** | | | | **Adjusted for team size** | | | | |
| --- | --- | --- | --- | --- | --- | --- | --- | --- | --- | --- | --- | --- | --- | --- | --- | --- | --- | --- | --- | --- |
| **Category/Item** | **dif** | **low** | **upp** | **p** | **dif** | **low** | **upp** | **p** | **dif** | **low** | **upp** | **p** | **dif** | **low** | **upp** | **p** | **dif** | **low** | **upp** | **p** |
| **Non-technical score*** | **27,2** | 10,5 | 43,9 | <0,1 | **26,4** | 9,4 | 43,4 | <0,1 | **28,0** | 10,0 | 46,0 | <0,1 | **29,1** | 12,5 | 45,7 | <0,1 | **23,8** | 6,3 | 41,4 | <0,1 |
| **1. Communication with patient** | **9,4** | -1,3 | 20,1 | 0,1 | **8,9** | -1,8 | 19,6 | 0,1 | **9,4** | -1,3 | 20,2 | 0,1 | **9,7** | -0,8 | 20,3 | 0,1 | **7,1** | -3,9 | 18,2 | 0,2 |
| 1.1 Information sharing | **10,1** | -1,6 | 21,8 | 0,1 | **11,2** | -0,5 | 23,0 | 0,1 | **10,5** | -1,3 | 22,3 | 0,1 | **9,8** | -1,8 | 21,4 | 0,1 | **7,2** | -5,1 | 19,5 | 0,2 |
| 1.2 Reassuring attitude | **8,3** | 1,7 | 14,9 | <0,1 | **8,3** | 1,7 | 14,9 | <0,1 | **8,2** | 1,5 | 14,9 | <0,1 | **8,1** | 1,5 | 14,7 | <0,1 | **7,6** | 1,0 | 14,2 | <0,1 |
| 1.3 Partner management | **3,1** | -5,9 | 12,2 | 0,5 | **2,9** | -6,2 | 12,0 | 0,5 | **3,0** | -6,2 | 12,1 | 0,5 | **3,6** | -5,4 | 12,6 | 0,4 | **1,0** | -8,3 | 10,3 | 0,8 |
| **2. Task/Case management** | **30,8** | 15,1 | 46,5 | <0,1 | **30,3** | 14,3 | 46,2 | <0,1 | **31,3** | 15,0 | 47,7 | <0,1 | **32,8** | 17,5 | 48,1 | <0,1 | **28,6** | 12,9 | 44,3 | <0,1 |
| 2.1 Plan of action | **11,2** | 4,3 | 18,1 | <0,1 | **11,0** | 4,1 | 18,0 | <0,1 | **11,2** | 4,1 | 18,2 | <0,1 | **11,8** | 5,0 | 18,5 | <0,1 | **10,0** | 2,9 | 17,1 | <0,1 |
| 2.2 Resources utilization | **11,6** | 2,5 | 20,8 | <0,1 | **11,2** | 1,9 | 20,6 | <0,1 | **11,5** | 2,2 | 20,8 | <0,1 | **12,0** | 3,1 | 20,9 | <0,1 | **12,2** | 3,3 | 21,2 | <0,1 |
| 2.3 Problem solving | **NA** | NA | NA | NA | **NA** | NA | NA | NA | **NA** | NA | NA | NA | **NA** | NA | NA | NA | **NA** | NA | NA | NA |
| **3. Teamwork** | **17,7** | 6,1 | 29,4 | <0,1 | **18,5** | 6,8 | 30,1 | <0,1 | **17,6** | 5,7 | 29,6 | <0,1 | **16,9** | 5,1 | 28,7 | <0,1 | **16,0** | 4,1 | 28,0 | <0,1 |
| 3.1 Leadership | **9,6** | 2,4 | 16,7 | <0,1 | **10,8** | 3,6 | 17,9 | <0,1 | **9,4** | 2,1 | 16,8 | <0,1 | **8,8** | 1,6 | 16,1 | <0,1 | **8,7** | 1,2 | 16,2 | <0,1 |
| 3.2 Role assignment | **9,1** | 0,7 | 17,4 | <0,1 | **9,2** | 0,9 | 17,6 | <0,1 | **8,9** | 0,5 | 17,3 | <0,1 | **8,4** | 0,1 | 16,8 | <0,1 | **8,2** | -0,2 | 16,7 | 0,1 |
| 3.3 Team interaction | **23,4** | 11,7 | 35,1 | <0,1 | **23,1** | 11,3 | 34,9 | <0,1 | **23,8** | 11,6 | 35,9 | <0,1 | **23,5** | 12,0 | 35,0 | <0,1 | **21,4** | 9,6 | 33,2 | <0,1 |
| **4. Situation awareness** | **17,7** | 7,9 | 27,5 | <0,1 | **17,7** | 7,5 | 28,0 | <0,1 | **18,1** | 7,4 | 28,7 | <0,1 | **20,7** | 11,1 | 30,2 | <0,1 | **16,1** | 5,7 | 26,5 | <0,1 |
| 4.1 Anticipation | **16,5** | 8,6 | 24,4 | <0,1 | **16,3** | 8,2 | 24,4 | <0,1 | **16,4** | 8,3 | 24,4 | <0,1 | **19,2** | 11,6 | 26,8 | <0,1 | **15,7** | 7,8 | 23,7 | <0,1 |
| 4.2 Realizing limitations | **4,9** | -0,9 | 10,7 | 0,1 | **4,7** | -1,3 | 10,8 | <0,1 | **4,9** | -1,1 | 10,8 | <0,1 | **6,4** | 0,7 | 12,0 | <0,1 | **4,6** | -1,4 | 10,6 | 0,1 |
| 4.3 Avoiding fixation | **14,7** | 6,3 | 23,1 | <0,1 | **14,4** | 5,7 | 23,2 | <0,1 | **14,8** | 5,6 | 23,9 | <0,1 | **16,7** | 8,4 | 25,0 | <0,1 | **12,9** | 3,7 | 22,1 | <0,1 |
| 4.4 Responsiveness | **14,3** | 5,3 | 23,3 | <0,1 | **13,9** | 4,7 | 23,0 | <0,1 | **14,3** | 4,9 | 23,7 | <0,1 | **15,0** | 6,2 | 23,8 | <0,1 | **12,4** | 3,2 | 21,6 | <0,1 |
| 4.5 Vigilance | **9,5** | -4,1 | 23,2 | 0,2 | **9,4** | -4,3 | 23,2 | 0,2 | **8,7** | -5,1 | 22,5 | 0,2 | **9,1** | -4,4 | 22,5 | 0,2 | **8,6** | -5,1 | 22,4 | 0,2 |
| **5. Team communication** | **NA** | NA | NA | NA | **NA** | NA | NA | NA | **NA** | NA | NA | NA | **NA** | NA | NA | NA | **NA** | NA | NA | NA |
| 5.1 Focused communication | **15,3** | 1,3 | 29,3 | <0,1 | **15,2** | 1,3 | 29,1 | <0,1 | **15,2** | 0,8 | 29,6 | <0,1 | **14,4** | 0,3 | 28,4 | <0,1 | **13,0** | -1,4 | 27,4 | 0,1 |
| 5.2 Closing the loop | **NA** | NA | NA | NA | **NA** | NA | NA | NA | **NA** | NA | NA | NA | **NA** | NA | NA | NA | **NA** | NA | NA | NA |
| **6. Environment of the room** | **4,7** | -11,2 | 20,7 | 0,6 | **3,2** | -12,9 | 19,3 | 0,7 | **4,6** | -11,6 | 20,7 | 0,6 | **4,1** | -11,9 | 20,1 | 0,6 | **4,1** | -12,0 | 20,2 | 0,6 |
| 6.1 Management of disruption | **NA** | NA | NA | NA | **NA** | NA | NA | NA | **NA** | NA | NA | NA | **NA** | NA | NA | NA | **NA** | NA | NA | NA |
| 6.2 Atmosphere in the room | **11,3** | 4,7 | 17,8 | 0,0 | **11,1** | 4,5 | 17,7 | <0,1 | **11,2** | 4,5 | 17,9 | <0,1 | **10,6** | 4,0 | 17,3 | <0,1 | **10,0** | 3,2 | 16,8 | <0,1 |

Data represents the scores from 60 teams managing vacuum extraction. Dif: Mean difference between non-technical score 2 to 5.

Low: lower 95% confidence interval, Upp: Upper 95% CI confidence interval.

*Represents the average ATOP score = (sum of 18 items score/18)
NA, non-applicable due to the distribution of data
